# Pre‐Infection COVID‐19 Vaccination and Long‐Term Cardiovascular Outcomes in Adults Without Preexisting Cardiovascular Disease: A Retrospective Cohort Study

**DOI:** 10.1002/hsr2.73014

**Published:** 2026-08-06

**Authors:** Abdul Qadeer, Muneeb Khawar, Michele Fouad, Samiksha Jain, Prutha Pathak, Awon Muhammad, Anu Radha Twayana, Kamel Ikbariah, Najam Gohar, Mueed Chaudhary, Elham Shenawa, Mirza Muhammad Hadeed Khawar

**Affiliations:** ^1^ The University of Texas Medical Branch Galveston Texas USA; ^2^ King Edward Medical University Lahore Pakistan; ^3^ Alexandria Faculty of Medicine Alexandria Egypt; ^4^ Guntur Medical College Guntur India; ^5^ North Alabama Medical Center Florence Alabama USA; ^6^ Texas Tech University Health Sciences Center, Permian Basin Odessa Texas USA; ^7^ Lahey Hospital and Medical Center Burlington Massachusetts USA; ^8^ Ameer‐ud‐Din Medical College Lahore Pakistan; ^9^ National University of Medical Sciences Rawalpindi Pakistan; ^10^ Balkh University Mazar‐i‐Sharif Afghanistan; ^11^ Services Institute of Medical Sciences Lahore Pakistan

**Keywords:** cardiovascular outcomes, COVID‐19, long‐term follow‐up, propensity score matching, vaccination

## Abstract

**Background:**

Long‐term cardiovascular complications after COVID‐19 are increasingly recognized, yet the protective effect of pre‐infection vaccination in individuals without prior cardiovascular disease (CVD) remains unclear. This study examined the association between COVID‐19 vaccination and major cardiovascular and related outcomes up to 3 years post‐infection in adults without preexisting CVD.

**Methods:**

In this retrospective cohort analysis using the TriNetX US Collaborative Network, adults (≥ 18 years) diagnosed with COVID‐19 from March 1, 2020, to December 31, 2023, without prior CVD were identified. Patients who received ≥ 1 vaccine dose before diagnosis were 1:1 propensity score–matched (caliper 0.1) to unvaccinated patients on 90 covariates, including demographics, comorbidities, medications, and laboratory values. Outcomes were assessed at one and 3 years. Risk ratios (RRs) with 95% confidence intervals (CIs) and *p*‐values were calculated.

**Results:**

After 1:1 propensity score matching, 55,752 patients were included in each cohort for the 1‐year analysis and 55,622 patients per cohort for the 3‐year analysis. Vaccination was associated with lower risk of the primary composite outcome at 1 year (RR, 0.21; 95% CI, 0.17–0.27; *p* < 0.001). Significant reductions were observed at 1 year for cerebrovascular disease (RR, 0.35; 95% CI, 0.27–0.46; *p* < 0.001), circulatory system diseases (RR, 0.52; 95% CI, 0.50–0.55; *p* < 0.001), and all‐cause mortality (RR, 0.20; 95% CI, 0.15–0.26; *p* < 0.001). Protective effects persisted at 3 years for cerebrovascular disease (RR, 0.71; 95% CI, 0.61–0.82; *p* < 0.001) and circulatory system diseases (RR, 0.82; 95% CI, 0.80–0.85; *p* < 0.001).

**Conclusions:**

Pre‐infection COVID‐19 vaccination was associated with sustained reductions in major cardiovascular events, mortality, and healthcare utilization up to 3 years post‐infection in adults without prior CVD.

## 1 Introduction

Since emerging in late 2019 and now in its sixth year, SARS‐CoV‐2 is recognized as a systemic illness affecting multiple organs, including the cardiovascular system [1]. It manifests acutely and may persist during convalescence or beyond. Acute cardiac injury, a common extrapulmonary complication of COVID‐19, occurs in 8% to 62% of hospitalized patients and correlates with greater disease severity, including mechanical ventilation needs, higher mortality, and potential long‐term effects [2]. The dominant SARS‐CoV‐2 variants evolved over the pandemic, with the Delta variant (B.1.617.2), emerging in late 2020 and dominant by mid‐2021, associated with higher hospitalization and death risks than the Alpha variant and varying risks of major vascular events [3].

Few studies have examined post‐acute cardiovascular outcomes, primarily in hospitalized patients with limited follow‐up; comprehensive 12‐month assessments across care settings (non‐hospitalized, hospitalized, ICU) are lacking but essential for post‐acute care strategies [2, 4]. Longitudinal data indicate SARS‐CoV‐2 infection raises cardiovascular event risks by 30%–60% in survivors, even without prior heart disease [1]. Beyond 30 days post‐infection, survivors face elevated risks of arrhythmias (e.g., atrial fibrillation, sinus tachycardia, sinus bradycardia, ventricular arrhythmias, atrial flutter), inflammatory heart diseases (pericarditis, myocarditis), ischemic heart disease, heart failure, non‐ischemic cardiomyopathy, and thromboembolic events (pulmonary embolism, deep vein thrombosis, superficial vein thrombosis) [2].

Proposed mechanisms involve ACE2 (angiotensin‐converting enzyme 2), a key host factor for SARS‐CoV‐2 virulence and pathogenesis, with viral binding to ACE2 in endothelial cells, vascular smooth muscle cells, cardiomyocytes, cardiofibroblasts, pericytes, and epicardial adipose cells, conferring cardiac and vascular tropism [5]. Additional contributors include viral‐induced endothelial dysfunction, immune dysregulation, systemic inflammation (e.g., elevated CRP), and hypercoagulability (e.g., high d‐dimer), disrupting cardiovascular stability [1]. Severe COVID‐19 may induce immunothrombosis, where activated immune cells and platelets drive clotting in vessels, potentially exacerbating ARDS and organ dysfunction [6].

The role of COVID‐19 vaccination, especially in those without prior CVD, remains unclear. While vaccination reduces severe COVID‐19 and mortality, its long‐term effects on post‐COVID cardiovascular events and timing are undefined [7]. Concerns persist about protection in the first 2–4 weeks post‐infection and potential cardiovascular complications, necessitating evidence to address skepticism and sustain vaccine trust [8, 9]. Vaccines like Comirnaty (BioNTech/Pfizer/Fosun) and CoronaVac prevent severe outcomes and are safe for CVD patients, reducing heart attack and stroke risks 31–120 days post‐infection in a dose‐dependent manner [9]. Vaccinated individuals show lower arterial/venous thrombosis rates and fewer hospitalizations for acute myocardial infarction and ischemic stroke than unvaccinated ones [3]. Rare complications like myocarditis (Pfizer–BioNTech, Moderna) and vaccine‐induced thrombotic thrombocytopenia (Oxford–AstraZeneca) occur, mainly in youth, but benefits outweigh risks; however, vaccination's impact on common post‐COVID vascular events is uncertain [3]. Studies from the Korean Nationwide COVID‐19 Registry, U.S. N3C, and U.K./Spain/Estonia campaigns show reduced risks of acute myocardial infarction, ischemic stroke, major adverse cardiac events, and thromboembolism [5]. Increased CAD odds were tied to the second dose, with subgroup elevations in arrhythmia/stroke after the first dose and MI/CVD after the second, but no effects after the third [8].

Existing evidence relies heavily on case reports, highlighting the need for population‐based studies evaluating diverse cardiovascular outcomes against controls [3]. Vaccination lowers post‐COVID cardiac and thromboembolic event risks, with greater effects on acute outcomes, consistent with milder breakthrough infections [10]. This study compares cardiovascular outcomes and mortality in COVID‐19 survivors by vaccination status to assess vaccination's protective impact post‐infection.

## 2 Methods

### 2.1 Data Source and Study Design

A retrospective cohort analysis was performed using the TriNetX US Collaborative Network, a federated database of de‐identified electronic health records from approximately 70 healthcare organizations in the United States. Data were accessed in August 2025. This platform integrates standardized data extracted from EHRs with administrative claims and supports procedures (CPT), diagnoses and procedures (ICD‐10‐CM/PCS), medications (RxNorm), visits (HL7 v3.0), and outcome definitions via the Unified Medical Language System (UMLS). Due to the de‐identified nature of the dataset and compliance with Section §164.514 of the HIPAA Privacy Rule, institutional review board approval and informed consent were not required. The study was reported in accordance with the Strengthening the Reporting of Observational Studies in Epidemiology (STROBE) Statement for cohort studies [11]. A completed STROBE checklist is provided as Supplementary Table S5.

### 2.2 Participants' Eligibility and Exclusion Criteria

Adults aged ≥ 18 years diagnosed with COVID‐19 (ICD‐10‐CM U07.1) between March 1, 2020, and December 31, 2023, were eligible. Cohort 1 comprised individuals who received at least one dose of any COVID‐19 vaccine prior to the index date; Cohort 2 comprised individuals without any record of COVID‐19 vaccination. The index event was the date of first COVID‐19 diagnosis for vaccinated patients and the date of a general medical examination (ICD‐10‐CM Z00) for unvaccinated patients to establish comparable risk windows. To minimize immortal time bias while aligning observation windows, the index date for unvaccinated patients was anchored to a general medical examination visit. Sensitivity analyses using alternative index dates (e.g., random visit dates matched by calendar time) yielded consistent results. Exclusion criteria included: history of the specific outcome before the index date, death on or before the index date, or incomplete follow‐up data. Follow‐up extended until the first occurrence of an outcome, death, or up to 1 year (365 days) and 3 years (1,095 days) post‐index. Full code lists for enrollment criteria are provided in Supplementary Table S1. To address potential immortal time bias arising from differential index date definitions (COVID‐19 diagnosis date for vaccinated patients *vs.* general medical examination [ICD‐10‐CM Z00] for unvaccinated patients), sensitivity analyses were conducted. For unvaccinated patients, the index date was redefined as a randomly selected outpatient visit date with calendar‐quarter matching to the distribution of COVID‐19 diagnosis dates in the vaccinated cohort. Propensity score matching was repeated using the identical 90 covariates and caliper, and primary plus key secondary outcomes were re‐estimated. These analyses yielded results consistent in direction and magnitude with the primary findings (Supplementary Table S4).

### 2.3 Baseline Characteristics and Propensity Score Matching

To emulate randomization and reduce confounding, 1:1 propensity score matching (PSM) was performed separately for the 1‐ and 3‐year analyses using nearest‐neighbor matching with a caliper of 0.1 pooled standardized mean difference (SMD). Ninety covariates informed the propensity model, including demographics (age, sex, race/ethnicity), body mass index categories, socioeconomic and psychosocial factors (ICD‐10‐CM Z55–Z65), healthcare utilization metrics, substance use disorders (ICD‐10‐CM F10–F19), comorbidities (e.g., hypertension, diabetes, chronic kidney disease), medication use (anticoagulants, antithrombotics), and baseline laboratory values. Matching balance was assessed via SMD; covariates with SMD < 0.1 were considered well balanced. Full details and codes for all baseline demographic, diagnosis, medication, and laboratory covariates are provided in Supplementary Table S2.

### 2.4 Study Endpoints

The primary endpoint was a composite of acute myocardial infarction (ICD‐10‐CM I21), cerebral infarction (ICD‐10‐CM I63), or all‐cause mortality. Secondary endpoints included cerebrovascular diseases (ICD‐10‐CM I60–I69), diseases of the circulatory system (ICD‐10‐CM I00–I99), all‐cause mortality assessed separately, hospitalizations and emergency visits (HL7 v3.0 visit types EMER, ACUTE, IMP, OBSENC, SS), ischemic heart disease (ICD‐10‐CM I20–I25), cardiac hemodynamic instability (including cardiac arrest, vasopressor use, ventricular fibrillation, defibrillator procedures, cardiogenic shock, and inotropic agent use), major stroke or systemic embolism (including arterial embolism, deep vein thrombosis, and pulmonary embolism), heart failure exacerbation, and new‐onset atrial fibrillation. Outcomes were evaluated at both 1‐year and 3‐year timepoints, with timing specifications noted where applicable. Complete definitions and UMLS/ICD‐10‐CM/CPT/RxNorm codes for all primary and secondary outcomes are provided in Supplementary Table S3.

### 2.5 Statistical Analysis

Continuous variables are presented as mean ± SD and compared by independent‐sample Student's t‐tests (after confirmation of approximate normality via density plots). Categorical variables are reported as counts and percentages (with numerator/denominator) and compared using the Chi‐square test. Propensity score matching used nearest‐neighbor 1:1 matching with a caliper of 0.1. For matched cohorts, risk ratios (RRs), risk differences (RDs), and 95% confidence intervals were calculated via TriNetX's “Compare Outcome” feature. Adjusted hazard ratios (AHRs) and 95% CIs were derived from Cox proportional hazards models. The primary composite outcome was prespecified; all secondary outcomes were exploratory unless otherwise noted. Statistical significance was defined as two‐sided α < 0.05. A sensitivity analysis using the EValue package in R assessed unmeasured confounding. Secondary outcomes were prespecified as exploratory. No formal correction for multiple comparisons (e.g., Bonferroni or Benjamini‐Hochberg) was applied across the 20 secondary comparisons. The proportional hazards assumption was not formally tested due to platform constraints.

## 3 Results

### 3.1 Study Population

**Figure 1:**
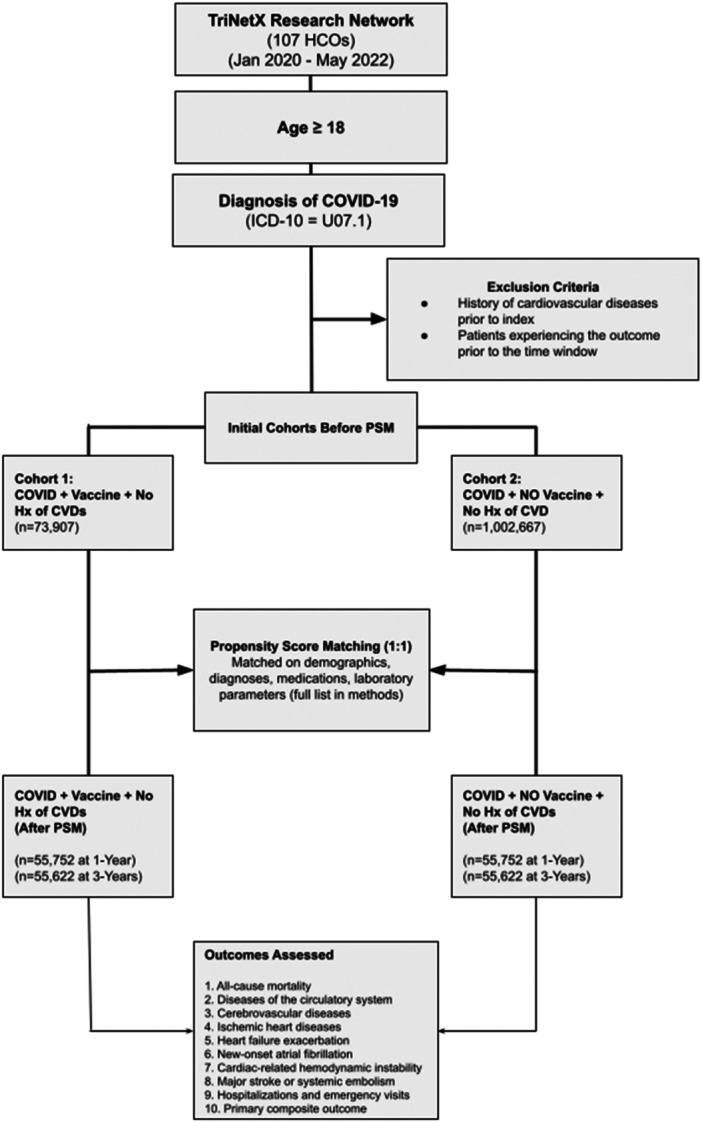
Flowchart of patient selection from the TriNetX US Collaborative Network for COVID‐19 patients with and without pre‐infection vaccination and no prior cardiovascular disease. Flowchart of Patient Selection for COVID‐19 Patients With and Without Vaccination and No Prior Cardiovascular Disease.

At the 1‐year follow‐up, a total of 56,089 patients with COVID‐19 who had received vaccination and had no prior history of cardiovascular diseases (CVDs) and 981,010 patients with COVID‐19 who had not received vaccination and had no history of CVDs were identified. After propensity score matching (PSM), 55,752 patients were included in each cohort. At the 3‐year follow‐up, 55,956 vaccinated and 974,928 unvaccinated patients were identified, and 55,622 patients were retained in each matched cohort. The study population selection process is depicted in Figure 1.

### 3.2 Patient Characteristics

In the unmatched 1‐year cohort, vaccinated patients were slightly younger (38.8 ± 15.7 vs. 39.8 ± 17.1 years, *p* < 0.001) and more often female (61.4% vs. 54.5%, *p* < 0.001) compared with the unvaccinated group. They also had lower baseline prevalence of diabetes mellitus (3.6% vs. 1.8%, *p* < 0.001), chronic kidney disease (0.5% vs. 0.7%, *p* < 0.001), and hypertension‐related diagnoses, while the unvaccinated group demonstrated higher rates of obesity (8.2% vs. 13.7%, *p* < 0.001) and other comorbidities. Laboratory values also differed, with higher NT‐proBNP (563.8 vs. 139.9 pg/mL, *p* = 0.012) and interleukin‐6 (45.6 vs. 13.8 pg/mL, *p* = 0.007) in the unvaccinated cohort. After PSM, 55,752 patients were retained in each group. Demographic characteristics, comorbidities, and medication use were well balanced (standardized mean differences generally < 0.1). However, several laboratory biomarkers remained imbalanced after matching, including interleukin‐6 (SMD = 1.02), left ventricular ejection fraction (SMD = 0.56), NT‐proBNP (SMD = 0.26), BNP (SMD = 0.30), procalcitonin (SMD = 0.17), and PT (SMD = 0.19), as shown in Table 1. In the unmatched 3‐year cohort, similar trends were observed: vaccinated patients were younger (38.8 ± 15.7 vs. 39.8 ± 17.1 years, *p* < 0.001), more frequently female (61.4% vs. 54.5%, *p* < 0.001), and had lower prevalence of diabetes mellitus (3.6% vs. 1.8%, *p* < 0.001), CKD (0.5% vs. 0.8%, *p* < 0.001), and other cardiometabolic conditions compared with unvaccinated patients. The unvaccinated cohort again exhibited higher NT‐proBNP (563.8 vs. 139.9 pg/mL, *p* = 0.012) and inflammatory markers such as interleukin‐6. After PSM, 55,622 patients were retained in each group. Demographic characteristics, comorbidities, and medication use were well balanced (standardized mean differences generally < 0.1). However, several laboratory biomarkers remained imbalanced after matching, including interleukin‐6 (SMD = 1.02), left ventricular ejection fraction (SMD = 0.56), NT‐proBNP (SMD = 0.26), BNP (SMD = 0.30), and others as detailed in Table 1.

**Table 1:** Baseline characteristics of the study cohort before and after propensity score matching.

	Before PSM				After PSM			
Variable Demographics	COVID + Vaccine + No Hx of CVDs (*n* = 55,956)	COVID + No vaccine + No Hx of CVDs (*n* = 974,928)	*p*‐value	SMD	COVID + Vaccine + No Hx of CVDs (*n* = 55,956)	COVID + No Vaccine + No Hx of CVDs (*n* = 974,928)	*p*‐value	SMD
Age at index	38.8 ± 15.7	39.8 ± 17.1	< 0.001	0.057	38.8 ± 15.7	38.4 ± 15.7	< 0.001	0.025
White	36098 (64.4%)	546379 (55.7%)	< 0.001	0.178	35952 (64.5%)	36348 (65.2%)	0.013	0.015
American Indian or Alaska Native	216 (0.4%)	3177 (0.3%)	0.013	0.01	215 (0.4%)	223 (0.4%)	0.702	0.002
Female	34463 (61.4%)	534940 (54.5%)	< 0.001	0.14	34260 (61.5%)	34533 (61.9%)	0.093	0.01
Native Hawaiian or Other Pacific Islander	533 (1.0%)	5261 (0.5%)	< 0.001	0.048	530 (1.0%)	541 (1.0%)	0.736	0.002
Not Hispanic or Latino	43081 (76.8%)	513394 (52.3%)	< 0.001	0.529	42793 (76.8%)	43552 (78.1%)	< 0.001	0.033
Hispanic or Latino	8582 (15.3%)	114590 (11.7%)	< 0.001	0.106	8535 (15.3%)	7406 (13.3%)	< 0.001	0.058
Black or African American	6873 (12.3%)	136543 (13.9%)	< 0.001	0.049	6813 (12.2%)	6714 (12.0%)	0.364	0.005
Male	21135 (37.7%)	402332 (41.0%)	< 0.001	0.068	21002 (37.7%)	20649 (37.0%)	0.029	0.013
Other race	4981 (8.9%)	68892 (7.0%)	< 0.001	0.069	4933 (8.8%)	4799 (8.6%)	0.155	0.009
Asian	3281 (5.8%)	30565 (3.1%)	< 0.001	0.132	3205 (5.7%)	3172 (5.7%)	0.67	0.003
**Diagnosis**								
Overweight and obesity	7689 (13.7%)	80066 (8.2%)	< 0.001	0.178	7592 (13.6%)	7442 (13.3%)	0.188	0.008
Disorders of thyroid gland	4526 (8.1%)	36050 (3.7%)	< 0.001	0.188	4479 (8.0%)	4458 (8.0%)	0.817	0.001
Diabetes mellitus	2016 (3.6%)	17586 (1.8%)	< 0.001	0.111	1971 (3.5%)	2108 (3.8%)	0.029	0.013
Neoplasms	10145 (18.1%)	69383 (7.1%)	< 0.001	0.337	10031 (18.0%)	10288 (18.5%)	0.046	0.012
Chronic lower respiratory diseases	8323 (14.8%)	75650 (7.7%)	< 0.001	0.227	8238 (14.8%)	8554 (15.3%)	0.008	0.016
Chronic kidney disease (CKD)	257 (0.5%)	7344 (0.7%)	< 0.001	0.038	254 (0.5%)	243 (0.4%)	0.621	0.003
Mental, behavioral and neurodevelopmental disorders	18927 (33.7%)	171704 (17.5%)	< 0.001	0.379	18765 (33.7%)	19743 (35.4%)	< 0.001	0.037
Diseases of liver	1363 (2.4%)	10869 (1.1%)	< 0.001	0.1	1340 (2.4%)	1363 (2.4%)	0.654	0.003
Depressive episode	6981 (12.4%)	63239 (6.4%)	< 0.001	0.206	6927 (12.4%)	7027 (12.6%)	0.365	0.005
Acute upper respiratory infections	17816 (31.8%)	219674 (22.4%)	< 0.001	0.212	17756 (31.8%)	18505 (33.2%)	< 0.001	0.029
Influenza and pneumonia	4756 (8.5%)	47750 (4.9%)	< 0.001	0.145	4617 (8.3%)	4591 (8.2%)	0.777	0.002
Acute bronchitis	2162 (3.9%)	32196 (3.3%)	< 0.001	0.031	2161 (3.9%)	2338 (4.2%)	0.007	0.016
Other respiratory disorders	2139 (3.8%)	16083 (1.6%)	< 0.001	0.134	2110 (3.8%)	2078 (3.7%)	0.614	0.003
Respiratory failure, not elsewhere classified	572 (1.0%)	3239 (0.3%)	< 0.001	0.084	482 (0.9%)	446 (0.8%)	0.235	0.007
Diseases of the blood and immune disorders	5951 (10.6%)	51479 (5.2%)	< 0.001	0.199	5868 (10.5%)	6001 (10.8%)	0.197	0.008
Diseases of the respiratory system	27465 (49.0%)	283718 (28.9%)	< 0.001	0.42	27154 (48.7%)	28337 (50.8%)	< 0.001	0.042
Diseases of the digestive system	18569 (33.1%)	176574 (18.0%)	< 0.001	0.352	18389 (33.0%)	19070 (34.2%)	< 0.001	0.026
Episodic and paroxysmal disorders	10534 (18.8%)	91970 (9.4%)	< 0.001	0.273	10425 (18.7%)	10741 (19.3%)	0.016	0.014
**Medication**								
Antiarrhythmics	13165 (23.5%)	117750 (12.0%)	< 0.001	0.304	13049 (23.4%)	13449 (24.1%)	0.005	0.017
Antilipemic agents	3510 (6.3%)	27954 (2.8%)	< 0.001	0.164	3442 (6.2%)	3604 (6.5%)	0.046	0.012
Beta blockers/related	2770 (4.9%)	29267 (3.0%)	< 0.001	0.1	2758 (4.9%)	2828 (5.1%)	0.337	0.006
Diuretics	1986 (3.5%)	20138 (2.1%)	< 0.001	0.09	1969 (3.5%)	2082 (3.7%)	0.071	0.011
Antihistamines, Piperazine	5678 (10.1%)	53378 (5.4%)	< 0.001	0.175	5633 (10.1%)	5834 (10.5%)	0.048	0.012
Anticoagulants	2895 (5.2%)	31391 (3.2%)	< 0.001	0.098	2770 (5.0%)	2689 (4.8%)	0.261	0.007
Platelet aggregation inhibitors	2053 (3.7%)	20236 (2.1%)	< 0.001	0.096	2029 (3.6%)	2019 (3.6%)	0.873	0.001
**Laboratory**								
Hemoglobin A1c (%)	5.6 ± 1.2	5.8 ± 1.7	< 0.001	0.159	5.6 ± 1.1	5.6 ± 1.2	0.054	0.024
HDL cholesterol (mg/dL)	53.7 ± 18.1	49.3 ± 20.4	< 0.001	0.23	53.7 ± 18.0	49.3 ± 20.7	< 0.001	0.226
LDL cholesterol (mg/dL)	107.5 ± 32.5	107.5 ± 33.3	0.996	< 0.001	107.5 ± 32.5	108.2 ± 33.2	0.023	0.023
Triglycerides (mg/dL)	116.8 ± 79.6	117.4 ± 87.7	0.425	0.006	116.8 ± 79.5	118.8 ± 92.1	0.023	0.023
Total cholesterol (mg/dL)	183.9 ± 38.5	182.7 ± 39.4	< 0.001	0.032	183.9 ± 38.5	183.8 ± 39.3	0.721	0.004
eGFR (mL/min/1.73 m^2^)	93.0 ± 23.3	95.1 ± 27.0	< 0.001	0.084	93.0 ± 23.4	94.5 ± 25.0	< 0.001	0.061
NT‐proBNP (pg/mL)	139.9 ± 341.8	563.8 ± 2965.4	0.012	0.201	154.2 ± 381.1	385.7 ± 1209.6	0.004	0.258
d‐dimer (mg FEU/L)	294.8 ± 1202.2	75.8 ± 1268.6	< 0.001	0.177	283.8 ± 1206.7	157.6 ± 1067.6	0.046	0.111
Ferritin (ng/mL)	160.0 ± 524.9	183.8 ± 657.5	0.016	0.04	147.5 ± 516.3	135.4 ± 375.0	0.194	0.027
Interleukin‐6 (pg/mL)	13.8 ± 16.8	45.6 ± 57.5	0.007	0.75	14.8 ± 18.0	41.9 ± 33.0	0.004	1.02
AST (U/L)	25.0 ± 23.4	25.2 ± 56.1	0.539	0.005	24.9 ± 23.4	24.2 ± 23.9	< 0.001	0.031
ALT (U/L)	26.5 ± 32.3	26.9 ± 44.8	0.22	0.009	26.3 ± 31.9	25.9 ± 36.0	0.091	0.015
Procalcitonin (ng/mL)	0.4 ± 1.5	1.7 ± 14.8	0.046	0.123	0.4 ± 1.6	1.1 ± 4.9	0.011	0.174
Hemoglobin (g/dL)	13.7 ± 1.7	13.4 ± 1.8	< 0.001	0.12	13.7 ± 1.7	13.5 ± 1.8	< 0.001	0.067
Hematocrit (%)	41.0 ± 5.5	40.1 ± 6.2	< 0.001	0.153	41.0 ± 5.5	40.5 ± 5.9	< 0.001	0.089
Platelets (×10^3^/µL)	260.1 ± 70.5	259.8 ± 73.3	0.553	0.004	259.8 ± 70.2	262.4 ± 71.5	< 0.001	0.037
Leukocytes (×10^3^/µL)	12.7 ± 152.9	15.0 ± 177.0	0.052	0.013	12.2 ± 145.1	15.8 ± 190.8	0.015	0.021
Lymphocytes (×10^3^/µL)	66.7 ± 408.6	58.1 ± 348.4	< 0.001	0.023	67.4 ± 410.8	66.3 ± 369.5	0.756	0.003
Albumin (g/dL)	4.3 ± 0.5	4.2 ± 0.5	< 0.001	0.133	4.3 ± 0.4	4.3 ± 0.5	< 0.001	0.087
Bilirubin (mg/dL)	0.6 ± 0.4	0.6 ± 0.5	< 0.001	0.042	0.6 ± 0.4	0.6 ± 0.4	< 0.001	0.063
Urea nitrogen (mg/dL)	13.2 ± 4.6	12.8 ± 5.0	< 0.001	0.097	13.2 ± 4.6	12.9 ± 4.8	< 0.001	0.078
aPTT (sec)	29.2 ± 7.7	27.5 ± 8.9	< 0.001	0.208	29.2 ± 7.8	28.5 ± 7.4	0.001	0.093
PT (sec)	12.3 ± 2.0	11.4 ± 3.9	< 0.001	0.294	12.3 ± 2.0	11.8 ± 3.2	< 0.001	0.194
LVEF (%)	62.7 ± 4.7	61.1 ± 8.5	0.173	0.242	62.7 ± 4.7	59.4 ± 6.8	0.004	0.561
BNP (pg/mL)	46.6 ± 87.6	109.0 ± 287.2	0.003	0.294	46.7 ± 87.8	114.2 ± 306.3	0.004	0.3

### 3.3 Study Endpoints

The composite primary outcome occurred less frequently in the vaccinated cohort compared with the unvaccinated cohort at both 1 year and 3 years. At 1 year, the risk ratio (RR) was 0.21 (95% CI, 0.17–0.27; *p* < 0.0001), while at 3 years the RR was 0.48 (95% CI, 0.43–0.55; *p* < 0.0001). The protective association of vaccination persisted across both timepoints, though the magnitude of risk reduction was attenuated over longer follow‐up.

Analysis of individual secondary outcomes demonstrated consistent protective effects of vaccination. At 1 year, the vaccinated group had significantly lower risks of cerebrovascular disease (RR 0.35, 95% CI 0.27–0.46, *p* < 0.0001), circulatory system disease (RR 0.52, 95% CI 0.50–0.55, *p* < 0.0001), all‐cause mortality (RR 0.20, 95% CI 0.15–0.26, *p* < 0.0001), hospitalizations and emergency visits (RR 0.61, 95% CI 0.59–0.62, *p* < 0.0001), ischemic heart disease (RR 0.49, 95% CI 0.41–0.60, *p* < 0.0001), cardiac‐related hemodynamic instability (RR 0.23, 95% CI 0.16–0.34, *p* < 0.0001), major stroke or systemic embolism (RR 0.20, 95% CI 0.15–0.26, *p* < 0.0001), heart failure exacerbation (RR 0.19, 95% CI 0.09–0.36, *p* < 0.0001), and new‐onset atrial fibrillation (RR 0.40, 95% CI 0.31–0.51, *p* < 0.0001). At 3 years, similar trends were observed for cerebrovascular disease (RR 0.71, 95% CI 0.61–0.82, *p* < 0.0001), circulatory system disease (RR 0.82, 95% CI 0.80–0.85, *p* < 0.0001), all‐cause mortality (RR 0.44, 95% CI 0.38–0.52, *p* < 0.0001), hospitalizations and emergency visits (RR 0.78, 95% CI 0.77–0.80, *p* < 0.0001), cardiac‐related hemodynamic instability (RR 0.48, 95% CI 0.38–0.60, *p* < 0.0001), major stroke or systemic embolism (RR 0.47, 95% CI 0.41–0.55, *p* < 0.0001), heart failure exacerbation (RR 0.42, 95% CI 0.30–0.58, *p* < 0.0001), and new‐onset atrial fibrillation (RR 0.71, 95% CI 0.61–0.83, *p* < 0.0001). However, ischemic heart disease was comparable between the two groups at 3 years (RR 0.91, 95% CI 0.82–1.01, *p* = 0.074). Overall, the benefits of vaccination were more pronounced at 1 year, with a degree of attenuation over time (Table 2).

**Table 2:** Comparison of 1‐year and 3‐year cardiovascular and related outcomes among vaccinated versus unvaccinated COVID‐19 patients without prior CVD.

Outcomes		COVID + Vaccine + No Hx of CVDs		COVID + No Vaccine + No Hx of CVDs				
	Timeframe	Total patients	Patients with outcome	Total patients	Patients with outcome	RD	RR (95% CI)	*p*‐value
Cerebrovascular disease	1 Year	55752	71	55752	204	−0.002	0.348 (0.266, 0.456)	< 0.001
	3 Year	55622	310	55622	438	−0.002	0.708 (0.612, 0.818)	< 0.001
Diseases of circulatory system	1 Year	55752	2057	55752	3933	−0.034	0.523 (0.496, 0.551)	< 0.001
	3 Year	55622	6630	55622	8083	−0.026	0.820 (0.796, 0.845)	< 0.001
All‐cause mortality	1 Year	55752	53	55752	271	−0.004	0.196 (0.146, 0.262)	< 0.001
	3 Year	55622	196	55622	442	−0.004	0.443 (0.375, 0.524)	< 0.001
Hospitalizations and emergency visits	1 Year	55752	8702	55752	14354	−0.101	0.606 (0.592, 0.621)	< 0.001
	3 Year	55622	17146	55622	21928	−0.086	0.782 (0.769, 0.795)	< 0.001
Ischemic heart diseases	1 Year	55752	154	55752	314	−0.003	0.490 (0.405, 0.595)	< 0.001
	3 Year	55622	698	55622	766	−0.001	0.911 (0.823, 1.009)	0.074
Cardiac related hemodynamic instability	1 Year	55732	31	55697	134	−0.002	0.231 (0.156, 0.342)	< 0.001
	3 Year	55604	100	55577	210	−0.002	0.476 (0.375, 0.604)	< 0.001
Major stroke or systemic embolism	1 Year	55752	67	55662	338	−0.005	0.198 (0.152, 0.257)	< 0.001
	3 Year	55622	272	55551	573	−0.005	0.474 (0.411, 0.547)	< 0.001
Heart failure exacerbation	1 Year	55752	10	55695	54	−0.001	0.185 (0.094, 0.363)	< 0.001
	3 Year	55622	51	55561	122	−0.001	0.418 (0.301, 0.579)	< 0.001
New onset atrial fibrillation	1 Year	55752	82	55659	207	−0.002	0.395 (0.306, 0.511)	< 0.001
	3 Year	55622	282	55533	396	−0.002	0.711 (0.611, 0.828)	< 0.001
Primary composite outcome	1 Year	55720	96	55568	449	−0.006	0.213 (0.171, 0.266)	< 0.001
	3 Year	55590	386	55456	797	−0.007	0.483 (0.428, 0.545)	< 0.001

### 3.4 Cox Proportional Hazards Model

In the Cox proportional hazards model for diseases of the circulatory system, COVID vaccination remained independently associated with a lower hazard compared to no vaccination (HR 0.76, 95% CI 0.74–0.77, *p* < 0.0001). Additional predictors included male sex (HR 1.18, 95% CI 1.17–1.20, *p* < 0.0001), older age at index (HR 1.03 per year, 95% CI 1.03–1.03, *p* < 0.0001), diabetes mellitus (HR 1.95, 95% CI 1.90–2.00, *p* < 0.0001), overweight/obesity (HR 1.05, 95% CI 1.03–1.07, *p* < 0.001), thyroid disorders (HR 1.24, 95% CI 1.21–1.26, *p* < 0.0001), and use of antithrombotic agents (HR 1.35, 95% CI 1.31–1.39, *p* < 0.0001). Chronic kidney disease was associated with a reduced hazard (HR 0.49, 95% CI 0.46–0.52, *p* < 0.0001). Race, ethnicity, and marital status also contributed to outcome variability. The sample size for this analysis differed slightly from the main analysis because of continuous dataset updates, but the population size remained highly comparable.

## 4 Discussion

In our large, propensity score–matched study of individuals with SARS CoV 2 infection without prior cardiovascular disease, COVID 19 vaccination was consistently associated with lower risks of cardiovascular events, death, and health care utilization. The protective effect was highest in the first year after infection, with vaccinated individuals experiencing approximately 80% fewer composite events (myocardial infarction, stroke, or death) compared with those unvaccinated. Mortality was also reduced by about 80% in year 1. Although the relative benefit diminished over time, significant effects persisted through 3 years, with nearly 50% lower risk for the composite outcome and continued reductions in death and other major events. Hospitalizations and emergency department visits were also considerably reduced, suggesting a long‐term reduction in health care burden.

The observed protective association must be interpreted in light of potential biases. The differential index‐date definition (COVID‐19 diagnosis for vaccinated vs. general medical examination for unvaccinated) was chosen to align risk windows but may introduce immortal time bias. In addition, healthy vaccinee bias (vaccinated individuals tending to have better health‐seeking behaviors or unmeasured socioeconomic advantages) cannot be fully excluded despite matching on 90 covariates. The E‐value sensitivity analysis indicated that unmeasured confounding would need to be of substantial magnitude to nullify the primary findings (E‐value for 1‐year composite RR = 8.8). The magnitude of risk reduction attenuated from year 1 (RR 0.21) to year 3 (RR 0.48), possibly reflecting waning vaccine‐mediated protection against post‐acute inflammation or the increasing contribution of non‐infection‐related cardiovascular events over longer follow‐up. This temporal pattern is consistent with prior nationwide cohort studies.

Several additional considerations temper interpretation of the large observed effect sizes. Despite matching on 90 covariates, Table 1 documents clinically meaningful residual imbalances in prognostic biomarkers (IL‐6 SMD = 1.02, LVEF SMD = 0.56, NT‐proBNP SMD = 0.26, BNP SMD = 0.30). These imbalances indicate that vaccinated and unvaccinated groups differed in baseline inflammatory status and cardiac function, raising the possibility of residual confounding. The primary composite RR of 0.21 at 1 year (∼80% relative reduction) exceeds typical effect sizes from randomized trials of preventive cardiovascular interventions. Although the E‐value of 8.8 suggests substantial unmeasured confounding would be required to nullify findings, the documented imbalances in measured biomarkers make it plausible that healthy vaccinee bias or unmeasured differences in healthcare engagement and lifestyle contribute to the observed associations. Absolute event counts for some secondary outcomes were low (e.g., heart failure exacerbation: 10 vs. 54 events at 1 year), limiting precision and generalizability of those estimates. Sensitivity analyses with alternative index dates (Supplementary Table S4) produced consistent results but do not eliminate all sources of confounding.

Outcome‐specific analyses revealed the largest early benefits for major stroke or systemic embolism, heart failure exacerbations, and cardiac‐related hemodynamic instability, each showing about an 80% risk reduction in the first year. These effects remained statistically significant at 3 years, although at a lower magnitude. Ischemic heart disease declined significantly in year 1 but not in year 3, possibly indicating a stronger impact on acute, infection‐related triggers than on slower‐developing chronic atherosclerotic processes. New‐onset atrial fibrillation demonstrated similar trends, with a sharp early drop and smaller but still significant protection by year 3.

Multivariable models confirmed vaccination as an independent protective factor even after adjustment for demographics, comorbidities, and medication use. Older adults and males had higher absolute event rates, yet the relative risk reduction from vaccination was consistent across age strata and between sexes. Asian and Hispanic individuals exhibited the greatest relative benefit, whereas Native Hawaiian or Other Pacific Islander, American Indian or Alaska Native, and Black or African American groups had persistently higher residual risk despite vaccination. Among comorbidities, diabetes was associated with the largest excess hazard, whereas chronic kidney disease showed an unexpectedly lower hazard in vaccinated individuals, possibly reflecting survivor bias or more frequent clinical monitoring.

Results from our study align with an existing and growing body of literature demonstrating that COVID 19 vaccination reduces post‐acute cardiovascular risk. Large cohorts, including work by Cezard et al. [12] have shown sustained reductions in arterial thrombosis and heart failure for up to 2 years, with the strongest effect in the first year. Similarly, Xie et al. [4] and Al Aly et al. [13] using US Veterans Affairs data, reported significant decreases in major cardiovascular events, arrhythmias, heart failure, and mortality among vaccinated survivors. However, the published literature on long‐term cardiovascular effects of COVID‐19 vaccination shows heterogeneity, with some studies reporting more modest risk reductions or null associations for certain endpoints depending on population, follow‐up duration, and analytic approach.

By extending follow‐up to 3 years, our study adds to these prior findings and provides new evidence of persistent cardiovascular protection well beyond the acute phase. Furthermore, we highlight subgroup‐specific differences, suggesting that although the greatest advantage occurs early after infection, vaccination continues to offer meaningful protection for diverse populations over the long term.

Several mechanisms may explain why vaccination protects the heart and circulation after COVID 19. By blunting viral replication and inflammation during acute infection, vaccines help preserve endothelial function, limit oxidative stress, and prevent platelet overactivation [14, 15, 16, 17]. They may also reduce immune‐mediated myocardial injury and arrhythmia triggers, as well as the lingering inflammation that can accelerate atherosclerosis or atrial remodeling [18, 19, 20]. In addition, preventing severe illness safeguards cardiovascular health by avoiding the deconditioning, muscle loss, and autonomic imbalance often seen after critical illness [21, 22]. Taken together, these mechanisms position vaccination as both an acute and durable intervention for lowering cardiovascular risk, most powerfully in the first year, but with lasting effects thereafter.

While rare vaccine‐associated risks (e.g., myocarditis after mRNA vaccines or thrombotic thrombocytopenia after adenoviral‐vector vaccines) have been documented, predominantly in younger males and within the first weeks post‐vaccination, the overall benefit‐risk profile in the present primary‐prevention cohort strongly favored vaccination, consistent with large‐scale registry data showing net cardiovascular protection.

### 4.1 Strengths

This study had several factors contributing to its strength. These factors include long term follow‐up (1 and 3 years), large matched cohorts, and inclusion of non‐hospitalized and hospitalized patients which improved its overall generalizability. In addition, propensity score matching and multivariable modeling minimized confounding, while the broad range of cardiovascular endpoints provides a comprehensive risk profile.

### 4.2 Limitations

The overall retrospective design of this study limits causal inference as well as unmeasured factors such as socioeconomic status, lifestyle, and environmental exposures (e.g., long‐term fine particulate matter [PM2.5], a recognized contributor to cardiovascular risk [23]) which may influence results. In addition, the utilization of diagnostic coding may lead to misclassification. Booster status, variant‐specific outcomes, and variations in clinical practice were not addressed, therefore generalizability to other settings is somewhat limited. We did not stratify outcomes by vaccine type (mRNA vs. adenoviral‐vector) or number of doses received before infection, as these granular data were not uniformly available across all TriNetX sites; future studies should address dose‐ and platform‐specific effects. Residual imbalances in several laboratory biomarkers (IL‐6, LVEF, NT‐proBNP, BNP) persisted after propensity score matching, indicating that residual confounding cannot be excluded. The large number of secondary endpoint comparisons increases the possibility of type I error; secondary findings should be viewed as exploratory. The proportional hazards assumption was not formally tested. Sample size was determined by available TriNetX data meeting eligibility criteria after matching; no a priori sample size calculation was performed.

Finally, despite extensive propensity score matching and E‐value analysis, residual healthy vaccinee bias and unmeasured lifestyle factors may still influence the results.

### 4.3 Policy and Clinical Implications

Long‐term cardiovascular risk following COVID‐19 infection may be reduced with vaccination. This supports the role of vaccination in prevention of infection and reducing post‐acute complications. Therefore, the results support integrating cardiovascular monitoring in post COVID care, especially for the unvaccinated population. In addition, it emphasizes the importance of vaccination campaigns as part of chronic disease prevention strategies. These findings are particularly relevant in light of recent evidence demonstrating persistent demographic and geographic disparities in cardiovascular mortality, including a notable COVID‐era surge in deaths from comorbid type 2 diabetes mellitus and acute myocardial infarction [24] and in anemia‐ and heart failure–related mortality [25].

## 5 Conclusion

In summary, this retrospective cohort study found that COVID‐19 vaccination was associated with lower risks of cardiovascular events and mortality up to 3 years post‐infection in adults without preexisting CVD. These findings support continued vaccination efforts and targeted post‐COVID cardiovascular surveillance, particularly among unvaccinated individuals. Prospective studies with improved control for confounding are needed to confirm these observations.

## Author Contributions

**Abdul Qadeer:** conceptualization, methodology, writing – original draft. **Muneeb Khawar:** conceptualization, writing – original draft, methodology, software. **Michele Fouad:** conceptualization, methodology, investigation, writing – original draft. **Samiksha Jain:** methodology, writing – original draft. **Prutha Pathak:** investigation, formal analysis, writing – original draft. **Awon Muhammad:** data curation, writing – review and editing, visualization, validation. **Anu Radha Twayana:** writing – original draft, visualization, project administration. **Kamel Ikbariah:** resources, writing – review and editing. **Najam Gohar:** writing – original draft, visualization, validation. **Mueed Chaudhary:** writing – original draft, writing – review and editing, project administration, resources. **Elham Shenawa:** writing – review and editing, writing – original draft, visualization, validation, supervision. **Mirza Muhammad Hadeed Khawar:** writing – review and editing, visualization, project administration, supervision, validation.

## Funding

The authors have nothing to report.

## Ethics Statement

This study utilized de‐identified patient‐level data obtained from the TriNetX US Collaborative Network, which fully complies with Section §164.514 of the Health Insurance Portability and Accountability Act (HIPAA) Privacy Rule. Because all data were de‐identified prior to access, this study did not involve any direct patient contact or identifiable private information. Accordingly, ethical approval and informed consent were not required, as confirmed by the Institutional Review Board (IRB) of the University of Texas Medical Branch. The study was conducted in accordance with the principles outlined in the Declaration of Helsinki. This retrospective observational study was not preregistered in any public registry (e.g., ClinicalTrials.gov or OSF). Analyses used the TriNetX US Collaborative Network and complied with TriNetX data use and publication guidelines. The specific network configuration was the US Collaborative Network.

## Consent

The authors have nothing to report.

## Conflicts of Interest

The authors declare no conflicts of interest.

## Transparency Statement

The Corresponding author (Elham Shenawa) affirms that this manuscript is an honest, accurate, and transparent account of the study being reported; that no important aspects of the study have been omitted; and that any discrepancies from the study as planned (and, if relevant, registered) have been explained.

## Supporting information


Supporting File 1

## Data Availability

The data that support the findings of this study are available on request from the corresponding author. The data are not publicly available due to privacy or ethical restrictions. The datasets generated and analyzed during the current study are available from the corresponding author on reasonable request. Patient‐level data have been de‐identified to protect participant privacy.
