# Supplementary material for: Pre‐Infection COVID‐19 Vaccination and Long‐Term Cardiovascular Outcomes in Adults Without Preexisting Cardiovascular Disease: A Retrospective Cohort Study
Source: Health Sci Rep. 2026 Aug 6;9(8):e73014. doi: 10.1002/hsr2.73014 (PMC13448144; doi:10.1002/hsr2.73014)
Supplement: Supplementary file 1 [file HSR2-9-e73014-s001.docx]

**Supplementary File**

| **Supplementary Table S1: Participants’ Eligibility with the relevant used codes.** | | | | | | | | | |
| --- | --- | --- | --- | --- | --- | --- | --- | --- | --- |
| COVID Vaccine (Cohort one) | | | | | No COVID Vaccine (Cohort Two) | | | | |
| Ungrouped Terms | | | | | Ungrouped Terms | | | | |
| Must have | | demographics | Age | Age at least 18 years (most recent occurrence) | Must have | | demographics | Age | Age at least 18 years (most recent occurrence) |
|  | | | | | Cannot have | | medication | NLM:CVX:213 | SARS-CoV-2 (COVID-19) Vaccine |
| Group 1 | | | | | Group 1 | | | | |
| Group 1A:  Date constraints: The terms in this group occurred between Jan 1, 2020 and May 1, 2022  Event Relationship: Any instance of COVID Vaccine occurred on or after any instance of COVID 19 | | | | | Group 1A:  Date constraint: The terms in this group occurred between Jan 1, 2020 and May 1, 2022  Event relationship: Any instance of No Hx of Cardiovascular Diseases occurred on or before the first instance of COVID-19 | | | | |
| Must have | | diagnosis | UMLS:ICD10CM:U07.1 | COVID-19 | Must have | | diagnosis | UMLS:ICD10CM:U07.1 | COVID-19 |
| Group 1B | | | | | Group 1B | | | | |
| Must have | | Medication | NLM:CVX:213 | SARS-CoV-2 (COVID-19) Vaccine | Cannot have |  | Diagnosis | UMLS:ICD10CM:I10-I15 | Hypertensive diseases (deprecated 2018) |
| Group 2 | | | | |  | or | Diagnosis | UMLS:ICD10CM:I10-I1A | Hypertensive diseases |
| Group 2A:  Date constraints: The terms in this group occurred at any time.  Event Relationship: Any instance of No Hx of CVDs occurred on or before any instance of COVID Vaccine | | | | |  | Or | procedure | UMLS:CPT:1012974 | Cardiovascular Procedures |
| Must have | | Medication | NLM:CVX:213 | SARS-CoV-2 (COVID-19) Vaccine |  | Or | diagnosis | UMLS:ICD10CM:I00-I99 | Diseases of the circulatory system |
| Group 2B | | | | |  | Or | Diagnosis | UMLS:ICD10CM:I00-I02 | Acute rheumatic fever |
| Cannot have |  | diagnosis | UMLS:ICD10CM:I10-I1A | Hypertensive diseases |  | Or | Diagnosis | UMLS:ICD10CM:I05-I09 | Chronic rheumatic heart diseases |
|  | or | diagnosis | UMLS:ICD10CM:I10-I15 | Hypertensive diseases (deprecated 2018) |  | or | Diagnosis | UMLS:ICD10CM:I26-I28 | Pulmonary heart disease and diseases of pulmonary circulation |
|  | Or | diagnosis | UMLS:ICD10CM:I30-I5A | Other forms of heart disease |  | Or | Diagnosis | UMLS:ICD10CM:I60-I69 | Cerebrovascular diseases |
|  | Or | diagnosis | UMLS:ICD10CM:I30-I52 | Other forms of heart disease (deprecated 2021) |  | Or | Diagnosis | UMLS:ICD10CM:I95-I99 | Other and unspecified disorders of the circulatory system |
|  | Or | diagnosis | UMLS:ICD10CM:I70-I79 | Diseases of arteries, arterioles and capillaries |  | Or | Diagnosis | UMLS:ICD10CM:I70-I79 | Diseases of arteries, arterioles and capillaries |
|  | Or | diagnosis | UMLS:ICD10CM:I80-I89 | Diseases of veins, lymphatic vessels and lymph nodes, not elsewhere classified |  | Or | Diagnosis | UMLS:ICD10CM:I20-I25 | Ischemic heart diseases |
|  | Or | diagnosis | UMLS:ICD10CM:I95-I99 | Other and unspecified disorders of the circulatory system |  | Or | Diagnosis | UMLS:ICD10CM:I80-I89 | Diseases of veins, lymphatic vessels and lymph nodes, not elsewhere classified |
|  | Or | diagnosis | UMLS:ICD10CM:I60-I69 | Cerebrovascular diseases |  | Or | Diagnosis | UMLS:ICD10CM:I30-I52 | Other forms of heart disease (deprecated 2021) |
|  | Or | diagnosis | UMLS:ICD10CM:I20-I25 | Ischemic heart diseases |  | Or | Diagnosis | UMLS:ICD10CM:I30-I5A | Other forms of heart disease |
|  | Or | diagnosis | UMLS:ICD10CM:I05-I09 | Chronic rheumatic heart diseases |  |  |  |  |  |
|  | or | diagnosis | UMLS:ICD10CM:I26-I28 | Pulmonary heart disease and diseases of pulmonary circulation |  |  |  |  |  |
|  | Or | diagnosis | UMLS:ICD10CM:I00-I02 | Acute rheumatic fever |  |  |  |  |  |
|  | Or | diagnosis | UMLS:CPT:1012974 | Cardiovascular Procedures |  |  |  |  |  |
|  | Or | diagnosis | UMLS:ICD10CM:I00-I99 | Diseases of the circulatory system |  |  |  |  |  |

| **Supplementary Table S2: Baseline Variables with their Relevant Codes** | | |
| --- | --- | --- |
| **Category** | **Code** | **Description** |
| **Demographics** | AI | Age at Index |
|  | 2106-3 | White |
|  | 1002-5 | American Indian or Alaska Native |
|  | F | Female |
|  | 2067-8 | Native Hawaiian or Other Pacific Islander |
|  | 2186-5 | Not Hispanic or Latino |
|  | 2135-2 | Hispanic or Latino |
|  | 2054-5 | Black or African American |
|  | M | Male |
|  | 2131-1 | Other Race |
|  | 2028-9 | Asian |
| **Diagnosis** | E66 | Overweight and obesity |
|  | E00-E07 | Disorders of thyroid gland |
|  | E08-E13 | Diabetes mellitus |
|  | C00-D49 | Neoplasms |
|  | J40-J4A | Chronic lower respiratory diseases |
|  | N18 | Chronic kidney disease (CKD) |
|  | F01-F99 | Mental, Behavioral and Neurodevelopmental disorders |
|  | K70-K77 | Diseases of liver |
|  | F32 | Depressive episode |
|  | J00-J06 | Acute upper respiratory infections |
|  | J09-J18 | Influenza and pneumonia |
|  | J20 | Acute bronchitis |
|  | J98 | Other respiratory disorders |
|  | J96 | Respiratory failure, not elsewhere classified |
|  | D50-D89 | Diseases of the blood and blood-forming organs and certain disorders involving the immune mechanism |
|  | J00-J99 | Diseases of the respiratory system |
|  | K00-K95 | Diseases of the digestive system |
|  | G40-G47 | Episodic and paroxysmal disorders |
| **Medications** | CV300 | ANTIARRHYTHMICS |
|  | CV350 | ANTILIPEMIC AGENTS |
|  | CV100 | BETA BLOCKERS/RELATED |
|  | CV700 | DIURETICS |
|  | AH105 | ANTIHISTAMINES, PIPERAZINE |
|  | BL110 | ANTICOAGULANTS |
|  | BL117 | PLATELET AGGREGATION INHIBITORS |
| **Laboratory** | 9037 | Hemoglobin A1c/Hemoglobin.total in Blood |
|  | 9001 | Cholesterol in HDL [Mass/volume] in Serum or Plasma |
|  | 9002 | Cholesterol in LDL [Mass/volume] in Serum or Plasma |
|  | 9004 | Triglyceride [Mass/volume] in Serum, Plasma or Blood |
|  | 9000 | Cholesterol [Mass/volume] in Serum or Plasma |
|  | 8001 | Glomerular filtration rate/1.73 sq M.predicted [Volume Rate/Area] in Serum, Plasma or Blood by Creatinine-based formula (MDRD) |
|  | 9072 | Natriuretic peptide.B prohormone N-Terminal [Mass/volume] in Serum, Plasma or Blood |
|  | 48065-7 | Fibrin D-dimer FEU [Mass/volume] in Platelet poor plasma |
|  | 9042 | Ferritin [Mass/volume] in Serum, Plasma or Blood |
|  | 26881-3 | Interleukin 6 [Mass/volume] in Serum or Plasma |
|  | 9047 | Aspartate aminotransferase [Enzymatic activity/volume] in Serum or Plasma |
|  | 9044 | Alanine aminotransferase [Enzymatic activity/volume] in Serum, Plasma or Blood |
|  | LG15749-1 | Procalcitonin [Mass/volume] in Serum, Plasma or Blood |
|  | 9014 | Hemoglobin [Mass/volume] in Blood |
|  | 9013 | Hematocrit [Volume Fraction] of Blood |
|  | 9020 | Platelets [#/volume] in Blood |
|  | 9015 | Leukocytes [#/volume] in Blood |
|  | LG32863-9 | Lymphocytes [#/volume] in Blood |
|  | 9045 | Albumin [Mass/volume] in Serum, Plasma or Blood |
|  | 9050 | Bilirubin.total [Mass/volume] in Serum, Plasma or Blood |
|  | 9030 | Urea nitrogen [Mass/volume] in Serum, Plasma or Blood |
|  | 9031 | Activated partial thromboplastin time (aPTT) in Plasma or Blood |
|  | 9033 | Prothrombin time (PT) in Plasma or Blood |
|  | 2003 | Left Ventricular Ejection Fraction (LVEF) (%) |
|  | 9003 | Natriuretic peptide B [Mass/volume] in Serum, Plasma or Blood |

| **Supplementary Table S3: Outcome definitions and Codes used for Identification** | | |
| --- | --- | --- |
| **Outcome** | **Code** | **Code Description** |
| Cerebrovascular Disease | UMLS:ICD10CM:I60-I69 | Cerebrovascular diseases |
| Diseases of circulatory system | UMLS:ICD10CM:I00-I99 | Diseases of circulatory system |
| All-Cause Mortality | Deceased | Deceased |
| Hospitalizations and Emergency visits | UMLS:HL7V3.0:VisitType:EMER | Visit: Emergency |
|  | UMLS:HL7V3.0:VisitType:ACUTE | Visit: Inpatient Acute |
|  | UMLS:HL7V3.0:VisitType:IMP | Visit: Inpatient Encounter |
|  | UMLS:HL7V3.0:VisitType:OBSENC | Visit: Observation Encounter |
|  | UMLS:HL7V3.0:VisitType:SS | Visit: Short Stay |
| Ischemic Heart Diseases | UMLS:ICD10CM:I20-I25 | Ischemic heart diseases |
| Cardiac related hemodynamic instability | UMLS:ICD10CM:I46 | Cardiac arrest |
|  | UMLS:ICD10PCS:3E030XZ | Introduction of Vasopressor into Peripheral Vein, Open Approach |
|  | UMLS:ICD10PCS:3E033XZ | Introduction of Vasopressor into Peripheral Vein, Percutaneous Approach |
|  | UMLS:ICD10PCS:3E040XZ | Introduction of Vasopressor into Central Vein, Open Approach |
|  | UMLS:ICD10PCS:3E043XZ | Introduction of Vasopressor into Central Vein, Percutaneous Approach |
|  | UMLS:ICD10PCS:3E050XZ | Introduction of Vasopressor into Peripheral Artery, Open Approach |
|  | UMLS:ICD10PCS:3E053XZ | Introduction of Vasopressor into Peripheral Artery, Percutaneous Approach |
|  | UMLS:ICD10PCS:3E060XZ | Introduction of Vasopressor into Central Artery, Open Approach |
|  | UMLS:ICD10PCS:3E063XZ | Introduction of Vasopressor into Central Artery, Percutaneous Approach |
|  | UMLS:ICD10CM:I49.01 | Ventricular fibrillation |
|  | UMLS:CPT:33249 | Insertion or replacement of permanent implantable defibrillator system, with transvenous lead(s), single or dual chamber |
|  | UMLS:CPT:33270 | Insertion or replacement of permanent subcutaneous implantable defibrillator system, with subcutaneous electrode, including defibrillation threshold evaluation, induction of arrhythmia, evaluation of sensing for arrhythmia termination, and programming or reprogramming of sensing or therapeutic parameters, when performed |
|  | UMLS:CPT:33271 | Insertion of subcutaneous implantable defibrillator electrode |
|  | UMLS:ICD10CM:R57.0 | Cardiogenic shock |
|  | NLM:RXNORM:3616 | dobutamine |
|  | NLM:RXNORM:52769 | milrinone |
|  | NLM:RXNORM:738 | inamrinone |
| Major Stroke or Systemic Embolism | UMLS:ICD10CM:I74 | Arterial embolism and thrombosis |
|  | UMLS:ICD10CM:I63 | Cerebral infarction |
|  | UMLS:ICD10CM:I82.409 | Acute embolism and thrombosis of unspecified deep veins of unspecified lower extremity |
|  | UMLS:ICD10CM:I26 | Pulmonary embolism |
| Heart Failure Exacerbation | UMLS:ICD10CM:I50.21 | Acute systolic (congestive) heart failure |
|  | UMLS:ICD10CM:I50.41 | Acute combined systolic (congestive) and diastolic (congestive) heart failure |
|  | UMLS:ICD10CM:I50.23 | Acute on chronic systolic (congestive) heart failure |
|  | UMLS:ICD10CM:I50.31 | Acute diastolic (congestive) heart failure |
|  | UMLS:ICD10CM:I50.33 | Acute on chronic diastolic (congestive) heart failure |
|  | UMLS:ICD10CM:I50.43 | Acute on chronic combined systolic (congestive) and diastolic (congestive) heart failure |
| New Onset Atrial Fibrillation | UMLS:ICD10CM:I48 | Atrial fibrillation and flutter |
| Primary composite outcome | UMLS:ICD10CM:I21 | Acute myocardial infarction |
|  | Deceased | Deceased |
|  | UMLS:ICD10CM:I63 | Cerebral infarction |
|  |  |  |

**Supplementary Table 4: Sensitivity Analysis Using Alternative Index Dates for the Unvaccinated Cohort (Random Visit Dates with Calendar-Quarter Matching)**

| **Outcome** | **Timeframe** | **COVID + Vaccine (n)** | **Events (Vaccine)** | **COVID + No Vaccine (n)** | **Events (No Vaccine)** | **RD** | **RR (95% CI)** | **p-value** |
| --- | --- | --- | --- | --- | --- | --- | --- | --- |
| Primary Composite Outcome | 1 Year | 53210 | 105 | 53210 | 478 | -0.007 | 0.220 (0.178–0.272) | <0.001 |
| Primary Composite Outcome | 3 Year | 53105 | 412 | 53105 | 825 | -0.008 | 0.499 (0.443–0.562) | <0.001 |
| Cerebrovascular Disease | 1 Year | 53210 | 78 | 53210 | 215 | -0.003 | 0.363 (0.280–0.470) | <0.001 |
| Cerebrovascular Disease | 3 Year | 53105 | 328 | 53105 | 455 | -0.002 | 0.721 (0.625–0.831) | <0.001 |
| Diseases of Circulatory System | 1 Year | 53210 | 2190 | 53210 | 4120 | -0.036 | 0.532 (0.505–0.560) | <0.001 |
| Diseases of Circulatory System | 3 Year | 53105 | 6890 | 53105 | 8350 | -0.027 | 0.825 (0.802–0.849) | <0.001 |
| All-Cause Mortality | 1 Year | 53210 | 58 | 53210 | 285 | -0.004 | 0.204 (0.154–0.270) | <0.001 |
| All-Cause Mortality | 3 Year | 53105 | 205 | 53105 | 455 | -0.005 | 0.451 (0.382–0.532) | <0.001 |
| Major Stroke or Systemic Embolism | 1 Year | 53210 | 72 | 53210 | 355 | -0.005 | 0.203 (0.157–0.262) | <0.001 |
| Cardiac Related Hemodynamic Instability | 1 Year | 53210 | 34 | 53210 | 142 | -0.002 | 0.239 (0.165–0.347) | <0.001 |

**Supplementary Table S5: STROBE checklist**

| **Item No** | **Recommendation** | **Reported on Page** | **Location / How Addressed** |
| --- | --- | --- | --- |
| **1a** | Indicate study design with commonly used term in title or abstract | Page 1 | Title + Abstract (Methods): “retrospective cohort analysis” stated |
| **1b** | Provide informative and balanced summary of what was done and what was found in abstract | Page 1 | Abstract (Background, Methods, Results, Conclusions) – includes design, matching, outcomes, RRs, CIs, and key findings |
| **2** | Explain scientific background and rationale | Page 2 | Introduction paragraphs 1–3 (COVID-19 cardiovascular complications, vaccination gap, mechanisms) |
| **3** | State specific objectives (including prespecified hypotheses) | Page 2 | End of Introduction + Abstract: association between pre-infection vaccination and long-term CV outcomes/mortality in adults without prior CVD |
| **4** | Present key elements of study design early | Page 3 | Methods 2.1: “A retrospective cohort analysis was performed using the TriNetX US Collaborative Network…” |
| **5** | Describe setting, locations, dates, recruitment, exposure, follow-up, data collection | Page 3 | Methods 2.1 & 2.2: TriNetX US Collaborative Network (≈70 HCOs), March 1 2020 – Dec 31 2023, 1-year & 3-year follow-up, de-identified EHR data |
| **6a** | Give eligibility criteria, sources and methods of selection, methods of follow-up | Page 3–4 + Supp | Methods 2.2 + Supplementary Table S1 (full eligibility codes, inclusion/exclusion, date constraints, event relationships) |
| **6b** | For matched studies: give matching criteria and number of exposed/unexposed | Page 4 + Page 5 | Methods 2.3 (1:1 PSM, caliper 0.1, 90 covariates) + Results 3.1 (n=55,752 per group at 1 year; n=55,622 per group at 3 years) + Table 1 |
| **7** | Clearly define all outcomes, exposures, predictors, potential confounders, effect modifiers + diagnostic criteria | Page 4 + Supp | Methods 2.3 (covariates) + 2.4 (endpoints) + Supplementary Table S2 (baseline codes) + Supplementary Table S3 (all outcome codes) |
| **8** | For each variable: sources of data, methods of assessment, comparability | Page 3–4 | Methods 2.1 (TriNetX standardized EHR/claims/CPT/ICD-10/RxNorm/UMLS/HL7), 2.3 (90 covariates), 2.4 (outcomes) |
| **9** | Describe efforts to address potential sources of bias | Page 3–4 + Page 7 | Methods 2.3 (PSM), 2.2 (sensitivity analyses for immortal time/index date), Discussion (healthy vaccinee bias, residual confounding, E-value) |
| **10** | Explain how study size was arrived at | Page 4 | Methods 2.5 (added): “Sample size was determined by available TriNetX data meeting eligibility criteria after matching; no a priori sample size calculation was performed” |
| **11** | Explain how quantitative variables were handled in analyses | Page 4–5 | Methods 2.5 + Results 3.2: continuous variables as mean ± SD; categorical as n (%); Table 1 reports both |
| **12a** | Describe all statistical methods, including those used to control for confounding | Page 4 | Methods 2.3 (1:1 nearest-neighbor PSM, caliper 0.1, SMD <0.1) + 2.5 (RR, RD, 95% CI via TriNetX; Cox models) |
| **12b** | Describe methods used to examine subgroups and interactions | Page 7 | Discussion (race/ethnicity, sex, age strata mentioned; Asian & Hispanic greatest relative benefit) |
| **12c** | Explain how missing data were addressed | Page 4 | Not explicitly detailed (TriNetX de-identified EHR platform limitation noted implicitly via platform description) |
| **12d** | If applicable, explain how loss to follow-up was addressed | Page 4 | Methods 2.2: “Follow-up extended until first occurrence of outcome, death, or up to 1/3 years”; censoring implicit in TriNetX outcome comparison |
| **12e** | Describe any sensitivity analyses | Page 4 + Supp | Methods 2.2 (detailed description of alternative index date sensitivity analyses) + Supplementary Table S4 (results) |
| **13a** | Report numbers at each stage (eligible, included, follow-up, analysed) | Page 5 | Results 3.1 + Figure 1 (flowchart): 56,089 vs 981,010 before matching → 55,752 per group after PSM (1-year); similar for 3-year |
| **13b** | Give reasons for non-participation at each stage | Page 5 | Results 3.1 + Figure 1 (exclusions: prior CVD, death before index, incomplete follow-up) |
| **13c** | Consider use of a flow diagram | Page 5 | Figure 1 (patient selection flowchart) present |
| **14a** | Give characteristics of study participants (demographic, clinical, social) and information on exposures and potential confounders | Page 5–6 | Results 3.2 + Table 1 (before/after PSM for demographics, diagnoses, medications, labs; 90 covariates listed) |
| **14b** | Indicate number of participants with missing data for each variable | Page 5–6 | Table 1 reports n for each variable; some lab variables have lower n (platform limitation) |
| **14c** | Summarise follow-up time | Page 3 + Page 5 | Methods 2.2 (maximum 365 days / 1,095 days) + Results 3.1 (separate 1-year and 3-year matched cohorts) |
| **15** | Report numbers of outcome events or summary measures over time | Page 6 | Table 2 (events, n, RD, RR, 95% CI, p-values for all outcomes at 1 and 3 years) |
| **16a** | Give unadjusted and confounder-adjusted estimates with precision; state which confounders adjusted for | Page 6 | Table 2 (PSM-adjusted RRs); Methods 2.3 lists the 90 covariates used for adjustment |
| **16b** | Report category boundaries when continuous variables categorized | Page 5–6 | Table 1 (age, labs reported continuously and categorically where relevant; no arbitrary cut-offs applied) |
| **16c** | If relevant, translate relative risk into absolute risk | Page 6 | Table 2 reports RD (absolute risk difference) alongside RR for all outcomes |
| **17** | Report other analyses (subgroups, interactions, sensitivity analyses) | Page 4 + Page 7 | Methods 2.2 & Supplementary Table S4 (sensitivity); Discussion (race/ethnicity subgroups, temporal patterns) |
| **18** | Summarise key results with reference to study objectives | Page 7 | Discussion opening paragraph + revised Conclusion (associative findings on CV events and mortality) |
| **19** | Discuss limitations taking into account sources of bias or imprecision; direction and magnitude | Page 7–8 | Dedicated Limitations paragraph (now expanded): immortal time, residual imbalances (IL-6, LVEF, NT-proBNP etc.), healthy vaccinee bias, low event counts, multiple comparisons, coding misclassification, US TriNetX only |
| **20** | Give cautious overall interpretation considering objectives, limitations, multiplicity, similar studies, other evidence | Page 7–8 | Discussion (mechanisms, comparison to literature, heterogeneity noted, E-value, residual confounding discussed) + revised Conclusion (associative language, call for prospective confirmation) |
| **21** | Discuss generalisability (external validity) | Page 7–8 | Discussion Strengths (large matched cohorts, hospitalized + non-hospitalized, long follow-up) + Limitations (TriNetX US Collaborative Network only, coding practices, variant/booster data unavailable) |
| **22** | Give source of funding and role of funders | Page 8 | Statements and Declarations – Funding & Acknowledgements (clarified: infrastructure support via UL1 TR001439 noted; no direct funding for this study) |
